# Willingness to adopt wearable devices with behavioral and economic incentives by health insurance wellness programs: results of a US cross-sectional survey with multiple consumer health vignettes

**DOI:** 10.1186/s12889-019-7920-9

**Published:** 2019-12-16

**Authors:** Diego Soliño-Fernandez, Alexander Ding, Esteban Bayro-Kaiser, Eric L. Ding

**Affiliations:** 1 Management Center Innsbruck, Innsbruck, Austria; 2 California Advanced Imaging, Novato, CA USA; 3 Institute for Artificial Intelligence, University of Bremen, Bremen, Germany; 4 Department of Nutrition, Harvard Chan School of Public Health, Boston, MA 02115 USA; 5 Microclinic International, San Francisco, CA USA; 6 Health Finance Institute, Arlington, VA USA; 7 Division of Health Analytics Solutions, General Dynamics Information Technology, Falls Church, VA USA

**Keywords:** Diffusion of innovation, Wearable electronic devices, Health insurance, United States

## Abstract

**Background:**

The number of health-related wearable devices is growing but it is not clear if Americans are willing to adopt health insurance wellness programs based on wearables and the incentives with which they would be more willing to adopt.

**Methods:**

In this cross-sectional study we used a survey methodology, usage vignettes and a dichotomous scale to examine U.S. residents’ willingness to adopt wearables (WTAW) in six use-cases where it was mandatory to use a wearable device and share the resulting data with a health insurance company. Each use-case was tested also for the influence of additional economic incentives on WTAW.

**Results:**

A total of 997 Americans across 46 states participated in the study. Most of them were 25 to 34 years old (40.22%), 57.27% were female, and 74.52% were white. On average, 69.5% of the respondents were willing to adopt health-insurance use-cases based on wearable devices, though 77.8% of them were concerned about issues related to economic benefits, data privacy and to a lesser extent, technological accuracy. WTAW was 11–18% higher among consumers in use-cases involving health promotion and disease prevention. Furthermore, additional economic incentives combined with wearables increased WTAW overall. Notably, financial incentives involving providing healthcare credits, insurance premium discount, and/or wellness product discounts had particularly greater effectiveness for increasing WTAW in the consumer use-cases involving participation: for health promotion (RR = 1.06 for financial incentive, 95% CI: 1.01–1.11; *P* = 0.018); for personalized products and services (RR = 1.11 for financial incentive, 95% CI: 1.01–1.21; P = 0.018); and for automated underwriting discount at annual renewal (RR = 1.28 for financial incentive, 95% CI: 1.20–1.37; *P* < 0.001).

**Conclusions:**

Under the adequate economic, data privacy and technical conditions, 2 out of 3 Americans would be willing to adopt health insurance wellness programs based on wearable devices, particularly if they have benefits related to health promotion and disease prevention, and particularly with financial incentives.

## Background

It is forecasted that by 2020, the number of connected wearable devices worldwide will surpass 600 million devices [1]. Even though, wearable devices are well-known for their fitness and wellness applications, they have the potential to improve population health by moving the focus from disease treatment to prevention; routinely monitoring personalized physiological measurements; supporting self-management; identifying alterations in health conditions; and creating positive long term behavioral changes towards healthy lifestyles [2–4]. Despite these opportunities, it is not clear if innovations based on wearable devices will be successfully implemented in the heath sector because, among other reasons, external forces such as key industry players, funding, public policy, technology, customers, and accountability can foster or kill wearable-based innovations [3, 5].

Health insurance companies play a central role among those external forces, particularly in the U.S. healthcare system where more than half of the population is covered by private employer-based group insurance [6]. In the case of health insurance, insights from wearable devices could lead to a more customer-centric model that anticipates customers’ risks and demands, and thus, enable new use cases related to continuous risk assessment; prediction of future health insurance claims; personalized insights to foster disease prevention; targeted marketing; customer engagement; and claims management [7–9].

In fact, wearables are gaining momentum in employer-based group insurance. For example, health insurance companies such as United Health Group, Humana, Cigna, and Highmark have established programs that foster the use of wearable devices at the workplace. In general terms, health insurance companies use wearable devices to promote wellness and prevention at the workplace and keep the progress accountable; and in exchange for healthy behaviors, employers receive economic incentives such as lower group premiums in their health insurance policy [10].

Previous studies have explained that health insurance companies could adjust their product strategies to motivate individuals to use wearable devices [2–4, 7–9, 11–15] and even evaluated if consumers were interested to use a wearable device and share the data with their health or life insurance company in return for financial rewards for healthy behaviors [16, 17]. However, little is known about the willingness to adopt health insurance wellness programs based on wearable devices, particularly those including use cases beyond health promotion. Therefore, the main goal of this study is to evaluate American’s willingness to adopt wearables (WTAW) with behavioral and economic incentives by health insurance wellness programs. An additional goal is to explore the influence of economic incentives on WTAW.

## Methods

### Population

In this cross-sectional study, we used Amazon Mechanical Turk as platform to obtain our study population. Previous studies show that Amazon Mechanical Turk can be used to obtain high-quality data that is as representative of the U.S. population, in terms of gender, age, race, and education, as traditional subject pools [18, 19]. Furthermore, it has been observed that participants from this online platform produce reliable results consistent with standard decision making biases [20].

Our target sample size was 1000 participants, so we posted two online surveys as a task in Amazon Mechanical Turk with a maximum limit of 500 respondents per survey. We randomized the opt-in process by posting both surveys the same day, and with the same title, description, and economic reward per response. Therefore, the two surveys had an almost identical positioning within Amazon’s Mechanical Turk and participants had no means to differentiate one from the other before entering the survey. Regarding inclusion and exclusion criteria, we included participants that were U.S. residents and at least 18 years old, and we excluded respondents who tried to fill-in the same survey more than once.

### Use-cases

We used a survey methodology based on vignettes to evaluate American’s WTAW by health insurance wellness programs. Given our research methods and data collection process, institutional review board deemed this research exempt of ethics assessment.

Regarding the use-cases presented in the vignettes of the surveys, they were all based on insights from scientific articles [2, 4, 13, 14]; reports about health insurance technology [3, 7–9]; and recent news about trends in health insurance innovation [10, 21, 22]. The six use-cases included were: health promotion, suggest actions to improve health status with a health assessment based on data from wearable devices; early detection of diseases, use data from wearable devices to identify certain diseases or disorders at an early stage; prediction of future health risks, use data from wearable devices to infer the likelihood of having a certain disease or disorder in the future; adherence tracking, wearable devices to identify specific movements such as smoking, drinking, eating or pill intake, and give actionable insights based on that data; personalized products and services, offer exclusive products and services related to wellness, health and insurance based on data from wearable devices; automated underwriting, speed up the process of applying or renewing the insurance policy by prefilling some fields with data from wearable devices. For additional information about the vignettes and the questions used in the surveys see Additional file 1.

### Willingness to adopt wearables

Each survey had two main sections. The first section consisted of demographic questions to better understand the characteristics of the sample in terms of age group; gender; income level; ethnicity; state of residence; marital status; type of health insurance; and employment status. The second section contained a set of six hypothetical use-cases. Each use-case presented a scenario in which a fictional health insurance policy holder named “Peter” had to choose whether to “Accept” or “Do not accept” a new health insurance service where it was mandatory to use a wearable device and share its data with a health insurance company. In every scenario, respondents had to select what they would do if they were Peter. If they chose “Accept” we considered they had a high WTAW in that specific use-case, whereas if they chose “Do not accept” we considered they had no WTAW.

To evaluate the main barriers to accept health insurance use-cases based on wearable devices, in addition to the dichotomous choice of accept or not accept, there was an “Accept just if …” option were respondents could select the conditions under which they would be willing to accept. These conditions included a free service; a free smart band; a service 100% accurate; data not shared with third-parties; and an “other” field where they could write their own requirements. If they chose “Accept just if …” we considered they were willing to accept that specific use-case but only under certain conditions.

### Influence of economic incentives

We created two surveys instead of one to test the influence of economic incentives on American’s WTAW. The only difference between surveys was that in one, the hypothetical use-cases included an economic incentive in their description while in the other they did not. Therefore, by comparing the results of the survey without economic incentive and the survey with an economic incentive we could analyze how economic incentives affect respondents’ choice to “Accept” or “Do not accept” a specific use-case. The economic incentives were not the same in the six use-cases, they were adapted to the specific context of each use-case to make the scenario as realistic as possible. Furthermore, the quantity of the economic incentives was not specified because, given the innovativeness of the use-cases, there was no clear benchmark in the health insurance market.

### Statistical analysis

We tabulated participant characteristics of the total sample (*n* = 1000) and by survey (*n* = 500). We visualized the main exposure of interest using three charts, two for the overall willingness to adopt (respondents who selected “Accept” and “Accept just if …” ) or not do adopt (respondents who selected “Do not accept”) wearables by type of use-case and by the absence or presence of economic incentive; and one for the main barriers to adopt wearables (conditions selected in “Accept just if …”).

Additionally, for the secondary outcome, we calculated the risk ratio of the WTAW in each use-case if there was an additional economic incentive and we considered a two-tailed *P* < 0.05 to be statistically significant. We used poisson regression with robust variance to estimate the Relative Risk of WTAW; we adjusted for the demographic variables age group, gender, education level, income level, ethnicity, health insurance, marital status, and employment status; and clustered on state of residence to correct for correlated observations within each state. We decided to use log-poisson with robust variances over logistic regression because leading epidemiologists and biostatisticians agree that the robust Poisson models are more robust to outliers compared to the log-binomial models when estimating relative risks or risk ratios for common binary outcomes; and odds ratio yields bias when outcome is not rare (i.e. > 5% yes) [23, 24]. Analyses used Stata software, version 13.1. (StataCorp LP, College Station, TX).

## Results

Out of the total sample, 3 participants submitted an empty survey, so they were excluded from the study, reducing the sample size to 997. In addition, 8 participants submitted the survey with at least one missing value. Given the relatively few missing values compared to the total sample size, missing data were handled through listwise deletion, meaning that we deleted all data from any participant with missing values. Regarding the general demographic characteristics of the sample, there were respondents from 46 states; 40.22% were 25 to 34 years old; 57.27% were female; 74.52% were white; 50.75% had private employer-based health insurance; 47.94% had some college or associate degree; and 20.60% had a household income of less than 25.000$ per year. For virtually all variables there were no statistically significant differences between the sample of the survey with economic incentive and the one without economic incentive. The only exception was geographic state and the subcategory of education level “some college or associate degree” which was overrepresented in the sample with economic incentive (46.89% vs 49.00%, *P* = 0.044). Table 1 shows the demographics characteristics of the participants.

**Table 1:** Demographic characteristics of the sample

Variable	Total sample (*n* = 997)
Gender	
Male	42.73% (*n* = 426)
Female	57.27% (*n* = 571)
Age group	
18–24 years old	13.14% (*n* = 131)
25–34 years old	40.22% (*n* = 401)
35–44 years old	22.47% (*n* = 224)
45–64 years old	21.26% (*n* = 212)
65 years or older	2.81% (*n* = 28)
Ethnicity	
White	74.52% (*n* = 743)
Hispanic or Latino	6.62% (*n* = 66)
Black or African American	8.93% (*n* = 89)
Asian American	7.62% (*n* = 76)
Other	1.81% (*n* = 18)
Missing	0.50 (*n* = 5)
Highest level of education attained	
Less than high school	0.90% (*n* = 9)
High school completion	11.13% (*n* = 111)
Some college or associate degree	47.94% (*n* = 478)
Advanced degree	39.62% (*n* = 395)
Missing	0.40% (*n* = 4)
Marital status	
Single, never married	43.93% (*n* = 438)
Married or domestic partnership	45.14% (*n* = 450)
Widowed	2.61% (*n* = 26)
Divorced	7.12% (*n* = 71)
Separated	0.80% (*n* = 8)
Missing	0.40% (*n* = 4)
Household income	
Less than 25.000$ per year	20.60% (*n* = 206)
Between 25.000$ and 34.999$ per year	13.44% (*n* = 134)
Between 35.000$ and 49.999$ per year	17.45% (*n* = 174)
Between 50.000$ and 74.999$ per year	23.87% (*n* = 238)
Between 75.000$ and 99.999$ per year	13.14% (*n* = 131)
Between 100.000$ and 149.999$ per year	9.03% (*n* = 90)
More than 150.000$ per year	2.21% (*n* = 22)
Missing	0.20% (*n* = 2)
Employment status	
Employed for wages	62.99% (*n* = 628)
Self-employed	13.74% (*n* = 137)
Unemployed	10.23% (*n* = 102)
Student	5.62% (*n* = 56)
Military	0.50% (*n* = 5)
Retired	3.71% (*n* = 37)
Unable to work	2.81% (*n* = 28)
Missing	0.40% (*n* = 4)
Health insurance	
Public (Medicare or Medicaid)	24.17% (*n* = 241)
Private (employer-based)	50.75% (*n* = 506)
Private (individually purchased)	11.74% (*n* = 117)
Other	3.01% (*n* = 30)
Not insured	9.53% (*n* = 95)
Missing	0.80% (*n* = 8)

**Fig. 1:**
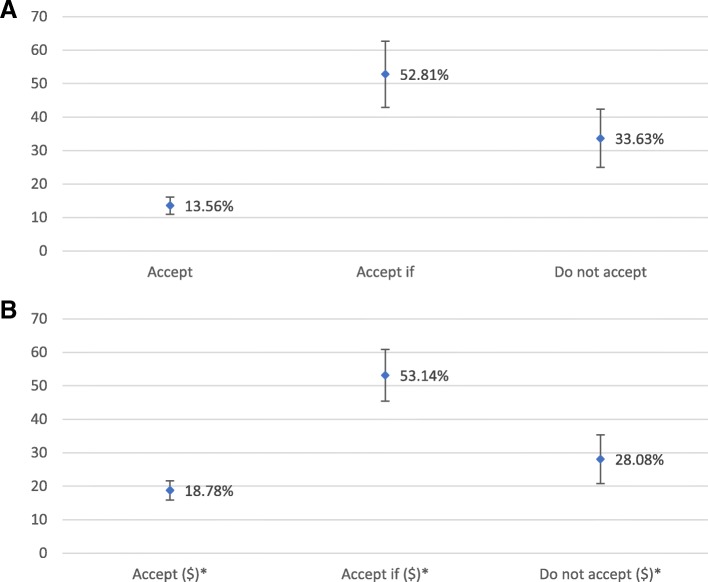
**a** Total distribution of average percentage of acceptance without economic incentive. **b** Total distribution of average percentage of acceptance with economic incentive

Figure 1a and b show the main results about participants’ WTAW by the presence or absence of an economic incentive. Results indicated that non-willingness to adopt dropped from 33.63% non-acceptance to 28.08% non-acceptance with economic incentives. Meanwhile, approximately 53% of the respondents were interested to conditionally accept if under certain use-cases conditions, almost unchanged by economic incentives. However, with an economic incentive, interest to unconditionally accept increased from 13.56 to 18.78%.

**Fig. 2:**
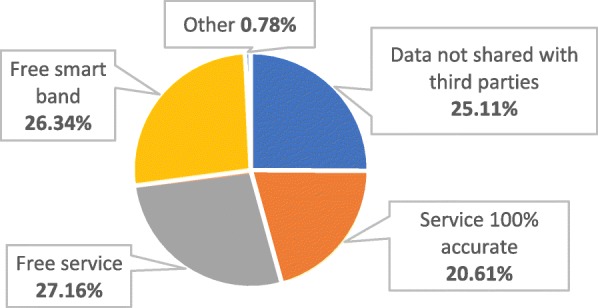
Main barriers to adoption by % of total conditional acceptances

Regarding American’s concerns about health-insurance use-cases based on wearable devices, Fig. 2 shows that out of the respondents who were interested to accept under certain conditions, 99.22% were concerned about economic benefits, data-privacy and/or technical conditions. More specifically, they placed a slightly higher importance to “free service”, “free smart band” and “data not shared with third parties” than to a “service 100% accurate” (27.16, 26.34 and 25.11% vs 20.61% respectively).

**Fig. 3:**
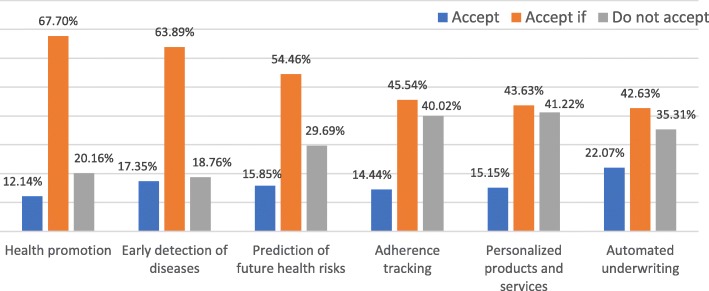
Willingness to adopt wearables in each use-case in %

Figure 3 shows that there were important differences in WTAW between use-cases, particularly among respondents who selected “Accept if” or “Do not accept”. Overall, Americans were more willing to adopt with and/or without conditions the use-cases of health promotion and early detection of diseases (79.84% and 81.24% respectively vs an average of 69.14%) and less willing to adopt the use-cases of adherence tracking, personalized products and services, and automated underwriting (59.98%, 58.78% and 64.70% respectively vs an average of 69.14%).

Regarding the inferential statistics, Table 2 shows the results of the influence of economic incentives on each of the use-cases based on data from wearable devices. Economic incentives involving providing healthcare credits, insurance premium discount, and/or wellness product discounts led to a significant increase in the WTAW in the use-cases of health promotion by 6% (RR=1.06 with P=0.018); personalized products and services by 11% (RR=1.11 with P=0.018); and automated underwriting by 28% (RR=1.28; with *P*<0.001). Furthermore, additional economic incentives had a suggestive finding for increasing the WTAW in the use-case of adherence tracking by 7% (RR=1.07 with *P*=0.05).

**Table 2:** Economic incentives on WTAW in each of the health insurance use-cases based on wearable device purpose of usage scenarios

Health insurance use-case based on wearable devices purpose of usage	RELATIVE RISK of WTAW with an additional economic incentive	*P* value	[95% Conf. Interval]	
Health promotion scenario	**1.06**	**0.018**	**1.01**	**1.11**
Early detection of diseases scenario	1.03	0.21	0.98	1.08
Prediction of future health risks scenario	0.78	0.27	0.51	1.21
Adherence tracking scenario	1.07	0.05	1.00	1.15
Personalized products scenario	**1.11**	**0.018**	**1.01**	**1.21**
Automated underwriting scenario	**1.28**	**<0.001**	**1.20**	**1.37**

Note. All models were adjusted for the demographic variables age group, gender, education level, income level, ethnicity, health insurance, marital status, and employment status; and clustered on state of residence to correct for correlated observations within each state

*P* values of <0.05, considered to be statistically significant, are presented in bold

## Discussion

Previous studies have explained that health insurance companies could adjust their product strategies to motivate individuals to use wearable devices [2–4, 7–9, 11–15] and even evaluated if consumers were interested to use a wearable device and share the data with their health or life insurance company in return for financial rewards for healthy behaviors [16, 17]. Other studies have examined the adoption of wearable devices as a whole [25] or by certain groups such as the elderly [26, 27], runners [11] or health professionals [28]; and found out that individuals follow a risk-benefit analysis too decide to adopt healthcare wearable devices [29].

Our study builds upon those findings and provides additional insights on users’ decision-making process and motivation by type of wearable-driven use-cases and contextual conditions. Even though, to the best of our knowledge, this is the first study to carefully examine individuals’ WTAW in specific health-insurance use-cases; if we assume that all use-cases consist on providing a health, financial, customization and/or convenience benefits to users in exchange of them using a wearable device and sharing the data with their health insurance company, some of the results could be compared with previous data; in fact, the results of this research are similar to the ones obtained by PriceWaterhouseCoopers in 2014 (69.14% vs 68% of respondents interested to adopt respectively) [17]. The study published by Life Happens and LIMRA in 2016 shows higher adoption rates (16.17% of respondents interested to adopt regardless of the conditions vs 30% of respondents very or extremely likely to adopt) but it is focused on life insurance, not health insurance [16]. Opposed to the study of Alley et all [25], our findings show that not only accuracy but also the financial cost of the wearable device is among the top barriers of the respondents, and that fewer citizens are not interested in using a wearable device (30.85% vs 44% respectively), but their research focused on the willingness to use a fitness tracker in general rather than specific wearable-driven use-case involving data-sharing with health insurance companies.

The main implications of our study are that, on the one hand, it ratifies that most Americans are willing to adopt wearable devices with behavioral and economic incentives by health insurance wellness programs, and on the other hand, it shows that not all use-cases are valued equally and that there are important barriers regarding economic, accuracy and data-privacy conditions. Another important finding is that Americans are more willing to adopt wearables if the use-cases focus on health benefits and/or there are additional economic incentives.

These insights are particularly relevant because the U.S. has signed into law the Republican Party’s repeal of a key provision of the Affordable Care Act (ACA), the individual mandate [30]. Among other changes, from 2019 onwards, people who have no health insurance and who are not exempt from the mandate under current law will not be penalized for not holding an active health insurance policy. Consequently, there is an expected exodus of young and healthy individuals from the health care marketplace, leaving sicker patients in the market for insurance. It is estimated that both the number of uninsured people could increase by 4 million in 2019, and average premiums in the ACA marketplaces could rise by approximately 10% [31].

This reform has a major impact because it creates adverse selection. As pointed out by the American Academy of Actuaries in a letter to Congress in March 2017, the sharpest decrease in insured rates would come from healthy individuals without immediate health care needs, and thus, the quality of the risk pool and health insurer’s ability to cover claims would diminish [32]. Therefore, health insurers will have even greater incentives to “cherry picking”, to attract young and healthy policy holders. That is especially relevant because wearables have been mostly purchased by individuals who have a healthy lifestyle [33] and the adoption of wearable devices tends to decline with age [34]. As a result, use-cases based on data from wearable devices could be potentially a very key incentive approach for health insurers to appeal to the younger and healthier segments of the population, and thereby retaining them to stabilize the insurance premiums in the risk pool. The results of this research can help to predict how Americans could react if health insurers offer wellness programs based on wearable devices, and thus, to better understand how the value of a use-case and its’ conditions could be modified to maximize the chances to attract or retain health insurance policy holders.

Given the exploratory nature of this research the results should be interpreted accordingly. This research cannot claim to be representative of the whole U.S. because there could be a sampling bias due to the relatively limited size of the sample and that there were no respondents from four states. Another limitation is that the order of the use-cases based on wearable devices was not randomized, and thus, there could be a question-order bias. In addition, the quantity of the economic incentives was not specified so it is not possible to conclude how substantial the economic incentives should be to increase American’s WTAW. Therefore, future research could address if the amount and type of economic incentive has a significant influence on the WTAW by health insurance wellness programs.

## Conclusion

Under the adequate economic, data privacy and technical conditions, two out of three Americans would be willing to accept health insurance wellness programs based on wearable devices, particularly if they have benefits related to health promotion and primary prevention. Therefore, health insurance wellness programs based on wearable devices could be potentially a very key incentive approach for health insurers to appeal to the younger and healthier segments of the population, and thereby retaining them to stabilize the insurance premiums in the risk pool.

## Supplementary information

**Additional file 1.** Surveys.

## Data Availability

The datasets used and/or analyzed during the current study are available from the corresponding author on reasonable request.

## Abbreviations

ACA: Affordable care act
WTAW: Willingness to adopt wearables
