# Supplementary material for: Willingness to adopt wearable devices with behavioral and economic incentives by health insurance wellness programs: results of a US cross-sectional survey with multiple consumer health vignettes
Source: BMC Public Health. 2019 Dec 16;19:1649. doi: 10.1186/s12889-019-7920-9 (PMC6912942; doi:10.1186/s12889-019-7920-9)
Supplement: Supplementary file 1 — Additional file 1. Surveys. [file 12889_2019_7920_MOESM1_ESM.docx]

ADDITIONAL FILE 1: Surveys

Both surveys can be accessed online (Survey 1, without economic incentive at https://goo.gl/forms/SZlvabClqpCGTlAM2 and Survey 2, with economic incentive at https://goo.gl/forms/yiJgds3YaKReaZdx2). Each use-case in Survey 1 contained the introductory paragraph plus option “a”, and each use-case in Survey 2 it contained the introductory paragraph plus option “b”. Except from the demographic questions, the main structure of the two surveys is presented below:

**Introductory paragraph:**

Peter’s health insurance company offered him a new service contingent on him using a new device, a smart wristband, and sharing the health data from his device with the insurance company.

1. **Use-case: Continuous health risk assessment and health promotion.**
2. *Health incentive:* The service consists in him receiving information about his current health risk (low, medium or high) and giving him individualized feedback so he can lower his health risk by, for example, being more active.
3. *Health and economic incentive:* (…) In addition, the cost of his health insurance would decrease if he lowers his health risk but not increase if he does not.
4. **Use-case: Early detection of diseases.**
5. *Health incentive:* The service consists in him receiving an alert if the analysis of the data from his wearable reveals a pattern related to a disease (e.g. hypertension or diabetes) or a disorder (e.g. anxiety or depression). This way he could go to the doctor in advance and prevent it.
6. *Health and economic incentive:* (…) In addition, those visits to the doctor would be free of charge or at a discounted price.
7. **Use-case: Prediction of future health risks.**
8. *Health incentive:* The service consists in him receiving a general prediction of his future health risk (low, medium or high) and information about how likely it is that he will have certain diseases (e.g. hypertension and diabetes) or disorders (e.g. anxiety and depression) in the future.
9. *Health and economic incentive:* (…) In addition, the cost of his health insurance would decrease if the prediction shows low risk but not increase if the prediction shows high risk.
10. **Use-case: Adherence tracking.**
11. *Health incentive:* The service consists in tracking daily movements such as smoking or pill intake and giving him actionable insights based on that data. That way he can know when and how much he smokes, or if he took his daily pills as recommended.
12. *Health and economic incentive:* (…) In addition, the cost of his health insurance premium would decrease if, for example, he is smoking less or taking all his medication but not increase if he is not smoking less or not taking all his medication.
13. **Use-case: Personalized products and services.**
14. *Customization incentive:* The service consists in him receiving individualized suggestions such as gym memberships, insurance products tailored to his lifestyle, and visits to nutritionists or other health professionals, based on his current health status.
15. *Customization and economic incentive:* (…) In addition, he would be able to access these products or use-cases at a discounted price.
16. **Use-case: Automated underwriting.**
17. *Convenience incentive:* The service consists in automating part of the process of applying or renewing the insurance policy, so Peter does not need to spend time filling every section of the application form.
18. *Convenience incentive and economic incentive:* In addition, Peter will receive monetary benefits either in health care credits or a discount on the cost of health insurance premium.

**QUESTION: What would you do if you were Peter?**

Accept.

Accept just if… (see next question)

Do not accept.

Accept just if… (select all that apply)

The use-case is for free.

The smart wristband is for free.

The use-case is always extremely accurate.

His data is not shared with third parties for commercial purposes

Other

**EXAMPLE:** Survey without economic incentive and the use-case of disease prevention.

Peter’s health insurer offered him a new use-case contingent on him using a smart wristband and sharing the health data from his device with the insurer. *The use-case consists on him receiving periodical feedback concerning his current health risk (low, medium or high) and giving him individualized insights so he can lower his health risk by adapting, for example, his fitness habits and sleep pattern.*
